# Correlation and agreement between eplet mismatches calculated using serological, low-intermediate and high resolution molecular human leukocyte antigen typing methods

**DOI:** 10.18632/oncotarget.24349

**Published:** 2018-02-01

**Authors:** Samantha Fidler, Lloyd D’Orsogna, Ashley B. Irish, Joshua R. Lewis, Germaine Wong, Wai H. Lim

**Affiliations:** ^1^ Department of Clinical Immunology, Fiona Stanley Hospital, Perth, Australia; ^2^ School of Pathology and Laboratory Medicine, University of Western Australia, Perth, Australia; ^3^ Department of Nephrology and Transplantation, Fiona Stanley Hospital, Perth, Australia; ^4^ School of Medicine and Pharmacology, University of Western Australia, Perth, Australia; ^5^ Centre for Kidney Research, The Children’s Hospital at Westmead, Sydney, Australia; ^6^ Sydney School of Public Health, University of Sydney, Sydney, Australia; ^7^ Centre for Transplant and Renal Research, Westmead Hospital, Sydney, Australia; ^8^ Department of Renal Medicine, Sir Charles Gairdner Hospital, Perth, Australia

**Keywords:** HLA typing, serological, molecular, agreement, Immunology section

## Abstract

Structural human leukocyte antigen (HLA) matching at the eplet level can be identified by HLAMatchmaker, which requires the entry of four-digit alleles. The aim of this study was to evaluate the agreement between eplet mismatches calculated by serological and two-digit typing methods compared to high-resolution four-digit typing. In a cohort of 264 donor/recipient pairs, the evaluation of measurement error was assessed using intra-class correlation to confirm the absolute agreement between the number of eplet mismatches at class I (HLA-A, -B, C) and II loci (HLA-DQ and -DR) calculated using serological or two-digit molecular typing compared to four-digit molecular typing methods. The proportion of donor/recipient pairs with a difference of >5 eplet mismatches between the HLA typing methods was also determined. Intra-class correlation coefficients between serological and four-digit molecular typing methods were 0.969 (95% confidence intervals [95% CI] 0.960–0.975) and 0.926 (95% CI 0.899–0.944), respectively; and 0.995 (95% CI 0.994–0.996) and 0.993 (95% CI 0.991–0.995), respectively between two-digit and four-digit molecular typing methods. The proportion of donor/recipient pairs with a difference of >5 eplet mismatches at class I and II loci was 4% and 16% for serological versus four-digit molecular typing methods, and 0% and 2% for two-digit versus four-digit molecular typing methods, respectively. In this small predominantly Caucasian population, compared with serology, there is a high level of agreement in the number of eplet mismatches calculated using two-compared to four-digit molecular HLA-typing methods, suggesting that two-digit typing may be sufficient in determining eplet mismatch load in kidney transplantation.

## INTRODUCTION

The human leukocyte antigen (HLA) system represents the loci of genes that determine tissue compatibility in solid organ transplantation. Over the last decade, HLA-typing has evolved from serological to molecular typing, which more precisely defines the immunological profiles of individuals, thereby providing a more comprehensive and accurate assessment of tissue compatibility in transplantation. Donor HLA alleles comprise multiple epitopes made up of polymorphic amino acid residues that can elicit a B-cell driven immune response in the recipients (immunogenicity), with a proportion of these epitopes capable of binding donor-specific anti-HLA antibody (antigenicity). Triplets and eplets are sequences of amino acid residues within each HLA epitope, which can elicit and bind to specific anti-HLA antibodies. [1]. Rene Duquesnoy first described triplets as linear sequences of three amino acid residues in antibody accessible positions on HLA molecules [1, 2]. Subsequently, eplets were defined as discontinuous amino acid residues within a 3 Ångstrom radius of a non-self residue [3]. These regions are considered a fundamental component of HLA epitopes recognized by anti-HLA antibodies. A large number of class I antibody-verified epitopes, corresponding to eplets or eplets paired with other residue configurations have been identified [4], and similar work to define class II eplet repertoires in HLA-DP, -DQ, -DR alleles are in progress [5]. Epidemiological studies have consistently shown that a greater number of eplet mismatches between the donor and recipient are associated with adverse graft outcomes after kidney transplantation, including development of de novo donor-specific anti-HLA antibodies [6, 7]. Consequently, eplet matching is now explicitly considered in several donor kidney allocation programs such as the Eurotransplant Acceptable Mismatch program and the Pediatric Renal Transplant program in Australia [8–10].

HLAMatchmaker is a computerized theoretical algorithm that calculates the number of eplet mismatches between donors and recipients by considering each HLA as a string of amino acid configurations in antibody-accessible positions [11–13]. Although four-digit HLA typing is required for the HLAMatchmaker program to calculate the number of eplet mismatches, two-digit typing can be converted into four-digit typing using the catalogue of common and well documented (CWD) alleles (version 2.0.0 of the CWD catalogue, available online at http://igdawg.org/cwd.html) and taking into account the allele frequency in a population or in a specific haplotype. Four-digit HLA typing methods are time consuming and expensive, and therefore not practicable in deceased donor kidney allocation whereby a rapid turnaround in HLA typing is essential. Consequently, intermediate resolution, two-digit molecular HLA typing methods and/or low resolution serological methods remain the standard typing technique in deceased donor kidney allocation. However, the correlation, reliability and agreement in the number of eplet mismatches calculated by serological or two-digit molecular compared to high-resolution four-digit molecular HLA typing methods remains unknown.

We aimed to determine the correlation, reliability and absolute agreement between currently available HLA typing methods to estimate the number of eplet mismatches between donors and recipients in kidney transplantation.

## RESULTS

Of the 264 donor/recipient pairs included in this study, 167 (63%) were deceased donor transplants, with 241 (91%), 15 (6%) and 8 (3%) recipients of caucasian, indigenous and asian ethnicity. There were no indigenous donors in this cohort. There were 164 (62%) male recipients with mean (SD) recipient age of 45 (11) years. Donor ethnicity was self-reported with 259 (98%) donors reported as caucasians.

The median (IQR) number of broad antigen HLA-mismatches at class I and II loci were 3 (2–5) and 3 (2–4), respectively; with median (IQR) calculated number of eplet mismatches at class I and II loci of 15 (9–23) and 19 (8–28), respectively for serological HLA typing method; 15 (10–22) and 19 (10–28), respectively for two digit molecular typing method; and 15 (15–22) and 19 (11–28), respectively for four digit molecular typing method.

### Consistency and absolute agreement between serological, two-digit molecular and four-digit molecular typing methods

Intra-class correlation coefficients with 95% CI for consistency and absolute agreement in the number of eplet mismatches calculated using serological or two-digit molecular and four-digit molecular typing methods are shown in Table 1. The consistency and absolute agreement in the number of eplet mismatches were similar between calculation using two-digit and four-digit molecular typing methods across both class I and II loci (Table 1). In contrast, the consistency and absolute agreement in the number of eplet mismatches were generally lower for serological typing method, particularly at HLA-C (consistency: 0.875 [95% CI 0.843, 0.900] and absolute agreement: 0.875 [95% CI 0.843, 0.900]) and HLA-DQ loci (consistency: 0.801 [95% CI 0.753, 0.840] and absolute agreement: 0.792 [95% CI 0.733, 0.837]) (Table 1). The closeness of the consistency and absolute agreement between the results of measurements suggests a high likelihood that the results are repeatable and reproducible.

**Table 1:** Intra-class correlation coefficients showing the consistency and absolute agreement between the calculated number of eplet mismatches estimated using two-digit molecular or serological human leukocyte antigen (HLA) typing compared to high-resolution four-digit molecular HLA typing (*n* = 264)

	Consistency^*^	Absolute agreement^*^
**Two-Digit**	**Four-Digit (95% CI)**	**Four-Digit (95% CI)**
**HLA-A**	0.998 (0.998–0.999)	0.998 (0.999–0.999)
**HLA-B**	0.993 (0.991–0.994)	0.993 (0.991–0.994)
**HLA-C**	0.989 (0.986–0.991)	0.989 (0.986–0.991)
**Class 1**	0.995 (0.994–0.996)	0.995 (0.994–0.996)
**HLA-DR**	0.989 (0.987–0.992)	0.989 (0.986–0.992)
**HLA-DQ**	0.984 (0.980–0.988)	0.984 (0.980–0.987)
**Class 2†**	0.994 (0.992–0.995)	0.993 (0.991–0.995)

	Consistency^*^	Absolute agreement^*^
**Serological**	**Four-Digit (95% CI)**	**Four-Digit (95% CI)**
**HLA-A**	0.995 (0.993–0.996)	0.995 (0.993–0.996)
**HLA-B**	0.983 (0.978–0.987)	0.982 (0.977–0.986)
**HLA-C**	0.875 (0.843–0.900)	0.875 (0.843–0.900)
**Class 1**	0.968 (0.960–0.975)	0.969 (0.960–0.975)
**HLA-DR**	0.980 (0.975–0.985)	0.980 (0.973–0.984)
**HLA-DQ**	0.801 (0.753–0.840)	0.792 (0.733–0.837)
**Class 2†**	0.931 (0.912–0.945)	0.926 (0.899–0.944)

^*^Two-way fixed intra-class correlation coefficients. ^†^Excludes HLA-DP.

**Figure 1:**
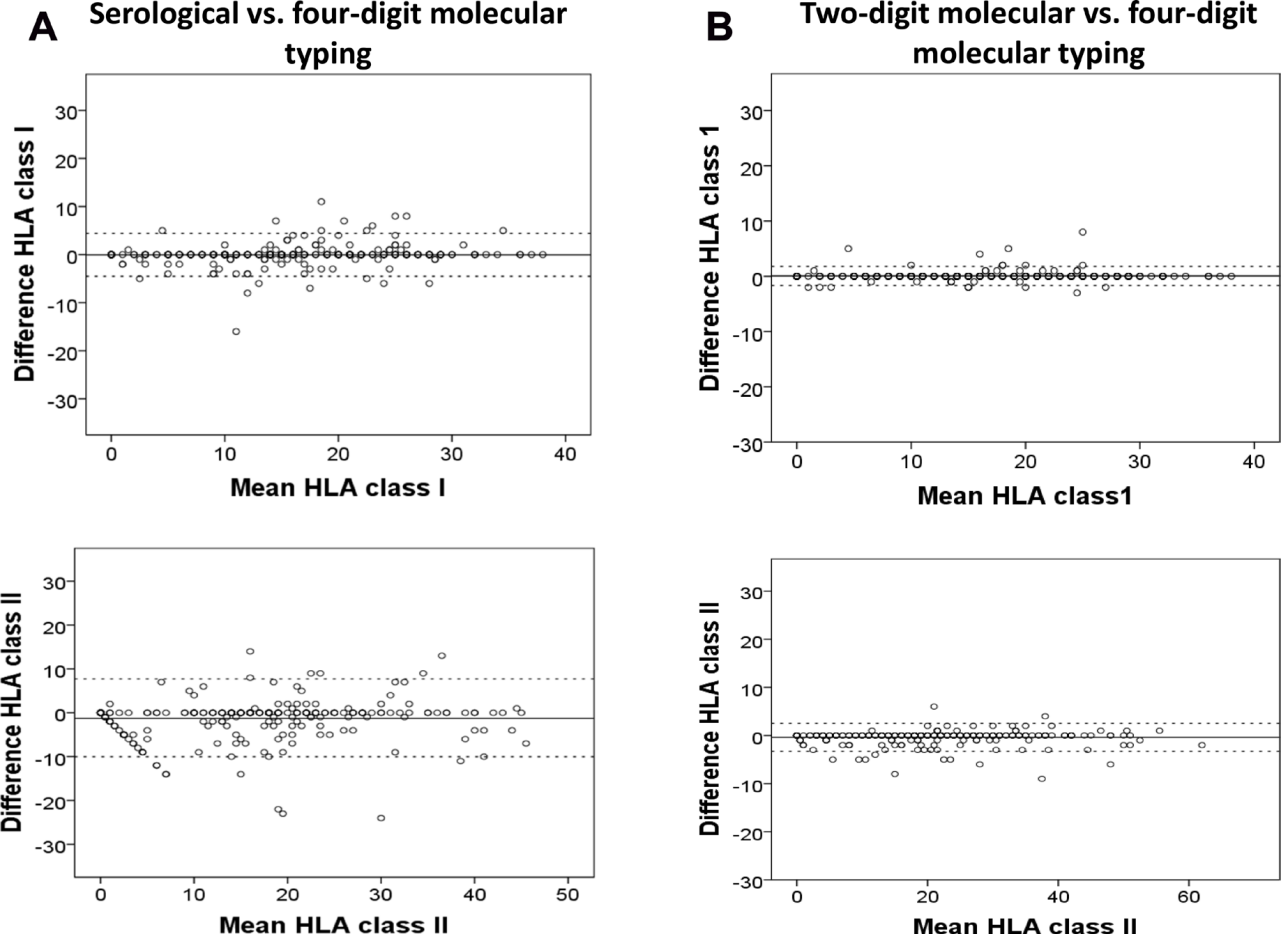
Bland-Altman plots showing the mean differences and 95% limits of agreements between the number of eplet mismatches at the class I (i.e. human leukocyte antigen [HLA]-A, -B and -C) and II loci (i.e. HLA-DR and -DQ, excluding HLA-DP) calculated by serological (A) or two-digit molecular HLA typing (B) compared to four-digit high-resolution molecular HLA typing methods (referent) Each open circle represents the estimated mean difference in the calculated number of eplet mismatches between serological or two-digit molecular typing and four-digit molecular typing methods for each donor/recipient pair in the cohort. The continuous line represents a mean difference of 0 eplet mismatch between the two HLA typing methods, with the discontinuous lines representing a mean difference of 1.96 standard deviations above and below a difference of 0 eplet mismatch.

The Bland-Altman plots of the mean differences and 95% limits of agreements between the number of eplet mismatches calculated by serological or two-digit molecular typing compared to four-digit molecular typing methods are shown in Figure 1 and Supplementary Figures 1 and 2. The narrow ranges between the two limits of agreement for class I and II eplet mismatches calculated using two-digit versus four-digit molecular typing methods indicates a strong agreement between the two HLA typing methods. For serological versus four digit molecular typing methods, the ranges between the two limits of agreement were wider indicating a lesser agreement in the number of eplet mismatches calculated by these two HLA typing methods.

### Magnitude of the differences in the number of eplet mismatches calculated using serological, two-digit molecular and four-digit molecular typing methods

Table 2 shows the proportion of donor/recipient pairs with a difference 0, 1–5, 6–10, 11–20 and >20 eplet mismatches between the three HLA typing methods at class I and II loci. For serological typing, the proportion of donor/recipient pairs where the calculated number of class I and II eplet mismatches were identical to the number of eplet mismatches calculated using four-digit molecular typing method was 71% and 58%, respectively. This compares with 88% and 73%, respectively for eplet mismatches calculated using two-digit molecular typing method. The proportion of donor/recipient pairs with a difference of six or more eplet mismatches between serological versus four-digit molecular typing methods at class I and II loci was 4% and 16%, respectively; compared to 0% and 2%, respectively between two-digit versus four-digit molecular typing methods. The most common scenario for donor/recipient pairs with a difference of six or more eplet mismatches between serological versus four-digit molecular HLA typing was the assignment of an incorrect DRB1 allele, thereby resulting in the erroneous assignment of HLA-DQ allele by linkage disequilibrium.

**Table 2:** Number (proportion) of donor recipient pairs with differences of 0, 1–5, 6–10, 11–20 and >20 eplet mismatches in the calculated number of class I and II eplet mismatches estimated using serological or two-digit molecular human leukocyte antigen (HLA) typing compared to high-resolution four-digit molecular HLA typing methods (*n* = 264)

	Difference between two-digit serological vs. four-digit molecular	Difference between two-digit molecular vs. four-digit molecular
**Class I eplet mismatches**		
**0**	187 (70.8)	231 (87.5)
**1–5**	66 (25.0)	32 (12.1)
**6–10**	10 (3.8)	0 (0.0)
**11–20**	1 (0.4)	0 (0.0)
**>20**	0 (0.0)	0 (0.0)
**Class II eplet mismatches**		
**0**	152 (57.6)	192 (72.7)
**1–5**	70 (26.5)	67 (25.4)
**6–10**	31 (11.7)	5 (1.9)
**11–20**	8 (3.0)	0 (0.0)
**>20**	3 (1.2)	0 (0.0)

Data expressed as number (%).

In the analysis restricted to indigenous recipients (*n* = 15), for serological typing, the proportion of donor/recipient pairs where the calculated number of class I and II eplet mismatches were identical to the number of eplet mismatches calculated using four-digit molecular typing method was 60% and 27%, respectively. This compares with 93% and 40%, respectively for eplet mismatches calculated using two-digit molecular typing method. The proportion of donor/recipient pairs with a difference of six or more eplet mismatches between serological versus four-digit molecular typing methods at class I and II loci was 13% and 20%, respectively; compared to 0% and 0%, respectively between two-digit versus four-digit molecular typing methods.

Of the 42 donor/recipient pairs with a difference of six or more eplet mismatches between serological compared to four-digit molecular typing methods at the class II locus, 18 (43%), 7 (17%) and 5 (12%) had differences of 0, 1–2 and >2 eplet mismatches at the class I locus. Of the 5 donor/recipient pairs with a difference of six or more eplet mismatches between two-digit compared to four-digit molecular typing methods at the class II locus, the calculated number of eplet mismatches at class I locus was identical in 4 pairs between the two typing methods. Table 3 shows the incorrect predicted four digit HLA alleles using two digit HLA alleles in donor/recipients pairs with a difference of six or more eplet mismatches between the two typing methods.

**Table 3:** Table showing the donor/recipient human leukocyte antigen (HLA) alleles that were misclassified using two digit molecular HLA typing compared to the actual four digit molecular HLA typing

	Two digit HLA typing	Predicted four digit HLA typing (from two digit)	Actual four digit HLA typing
**Class II (*n* = 5)**			
**Donor** **Recipient**	DRB1^*^07, ^*^03 DRB1^*^04	DRB1^*^07:01, ^*^03:01 DRB1^*^04:01	DRB1^*^07:01, 03:01 DRB1^*^04:02, ^*^04:03
**Donor** **Recipient**	DRB1^*^04, ^*^07 DRB1^*^04	DRB1^*^04:01, ^*^07:01 DRB1^*^04:01	DRB1^*^04:01, 07:01 DRB1^*^04:05, ^*^04:12
**Donor** **Recipient**	DQB1^*^03 DQB1^*^03	DQB1^*^03:01 DQB1^*^03:01	DQB1^*^03:01, ^*^03:02 DQB1^*^03:02
**Donor** **Recipient**	DQB1^*^03, ^*^06 DQB1^*^03, ^*^06	DQB1^*^03:01, ^*^06:04 DQB1^*^03:01, ^*^06:04	DQB1^*^03:01, ^*^06:04 DQB1^*^03:02, ^*^06:03
**Donor** **Recipient**	DQB1^*^03, ^*^05 DQB1^*^03, ^*^05	DQB1^*^03:01, ^*^05:01 DQB1^*^03:01, ^*^05:01	DQB1^*^03:01, ^*^05:01 DQB1^*^03:02, ^*^05:01

Only donor/recipient pairs with differences of at least 6 eplet mismatches between two digit and four digit molecular typing methods are shown.

## DISCUSSION

There was close agreement between the number of class I and II eplet mismatches calculated using two-digit molecular HLA typing method compared to four-digit molecular HLA typing method, with over 98% of the difference in the number of eplet mismatches within five or less from the gold standard. In contrast, there was poorer agreement between the number of eplet mismatches calculated using serological typing method compared to four-digit molecular typing method, particularly for class II eplet mismatches. This poorer agreement is not unexpected and is likely attributed to the incorrect assignment of HLA-C and -DQ antigens using linkage disequilibrium, which can lead to either over- or under-estimation of the true number of eplet mismatches between donors and recipients. If the number of donor/recipient eplet mismatches is explicitly considered in the deceased donor kidney allocation algorithm or decision-making process on whether to accept a donor kidney, under and over-estimating the number of eplet mismatches could potentially lead to erroneous assessment of immunological risk and may reduce the transplant potential, respectively. Even though two-digit HLA typing eliminates the incorrect assignment of HLA-C and HLA-DQ antigens by linkage disequilibrium, over 25% of the difference in the number of eplet mismatches were between one and five eplet mismatches from the gold standard, which could conceivably influence the transplant potential of patients in a donor allocation system that considers eplet matching (e.g. Eurotransplant Acceptable Mismatch program that considers 0–2 eplet mismatches as an acceptable mismatch). Given that the most common de novo donor-specific anti-HLA antibodies are directed against HLA-DQ, which are associated with the development of chronic antibody mediated rejection and transplant glomerulopathy, incorrect assignment of HLA-DQ allele using serological or two digit molecular HLA typing may lead to a more inaccurate assessment of immunological risk [7, 14]. Consequently, our findings suggest that two-digit typing across all HLA alleles should be the minimum requirement in the calculation of eplet mismatches, however clinicians will need to be aware of the uncertainty in the exact calculation of the number of eplet mismatches in all donor/recipient pairs.

HLA-mismatches remains the standard triage test for deceased donor kidney allocation because increasing number of HLA-A, -B and -DR mismatches are associated with an incremental risk of acute rejection and/or graft loss [15]. However, there is increasing evidence supporting the clinical importance of epitope or eplet matching in kidney transplantation [6, 16, 17]. Cohort studies have shown that HLA-locus-specific epitope or eplet mismatches are associated with the development of de novo donor-specific anti-HLA antibody, acute rejection or transplant glomerulopathy after kidney transplantation, all of which are associated with premature graft loss. In a study of 286 kidney transplant recipients, every incremental HLA-DR or HLA-DQ epitope mismatch was associated with an adjusted odds ratio of 1.06 and 1.04, respectively for developing de novo locus-specific anti-HLA antibody [6]. Similarly, in a case-control study of 156 kidney transplant recipients, each 10 additional eplet mismatches at HLA-DR and HLA-DQ was associated with a 25% greater risk of developing transplant glomerulopathy [7]. Eplet mismatches have also been shown to improve the risk stratification of the risk of acute rejection in kidney transplant recipients who have received 0–2 HLA-mismatched kidneys [18].

Incorporating an eplet based system for immunological assessment in clinical organ transplant allocation and/or determining transplant suitability is challenging. The development of next generation sequencing methods has revolutionized whole-genome analysis such that complete HLA allelic sequences, with resolution between two to eight-digit resolution, enables in-depth HLA gene sequencing with high precision and accuracy [19]. The additional expense and excess time associated with four-digit molecular HLA typing method compared to two-digit molecular typing method, is currently a significant impediment to its wider use in deceased donor allocation pathways, because of the short time frame available in deceased organ allocation. Even though the longer completion time for four digit HLA typing is not logistically feasible for deceased donor kidney allocation, it is possible and recommended that four digit typing be applied in the work-up for live-donor kidney transplant. However, in order to assess the potential benefit of incorporating eplet mismatches in the allocation of donor kidneys in anticipation of future opportunities for its wider use, evaluating the correlation and agreement between two-digit and four-digit molecular typing methods is extremely important and of clinical relevance. Even though next generation sequencing may eventually supersede Sanger sequencing technique, molecular typing using Sanger sequencing remains the standard typing technique in a large majority of tissue typing laboratories worldwide and therefore, the findings from this study will still be of relevance in current clinical practice.

It must be emphasized that a greater load of eplet mismatches merely increases the probability of adverse allograft outcomes and recent evidence suggest that identifying immunogenic eplets may improve the accuracy in predicting allograft outcomes after kidney transplantation. Production of HLA antibody may be influenced by one immunizing eplet, or may require an additional contact site at some distance from the primary eplet as demonstrated in the modelling of antibody interactions with HLA-DQ molecules. *Tambur et al.* has shown that whilst the immunizing eplet mismatch may occur on one chain of the DQ heterodimer, the contact sites for the antibody may potentially span across both the α and β chains [20]. A number of class I and II antibody-verified immunogenic epitopes/eplets have been identified and recorded in the epitope registry (http://www.epregistry.com.br) [4, 5], but this remains a work in progress. However, the association between the number and type (class I vs. II) of immunogenic eplet mismatches and allograft outcomes remains unclear. Our data support the view of Duquesnoy and colleagues who proposed that high resolution HLA typing should be used in the clinical setting to determine epitope mismatches [21]. This matching strategy has been used successfully for many years in selecting the most appropriate donor for highly sensitised patients in the Eurotransplant acceptable mismatch program and more recently a cohort of pediatric patients enrolled in the Australian paired kidney exchange program, which has led to improve transplant potential and/or selecting a better immunologically matched donor (i.e. with a lower number of eplet mismatches) for highly sensitized patients, therefore potentially avoiding or minimizing the risk of antibody mediated rejection and de novo production of donor-specific anti-HLA antibody after transplantation [10, 22].

Even though Sanger sequencing was used to determine the two-digit molecular HLA typing, the results of our study are applicable to any other two-digit typing platform. Rapid two-digit HLA typing of all loci can be obtained in under 90 minutes for quantitative polymerase chain reaction (qPCR) and under 3 hours for PCR-sequence-specific oligonucleotide (SSO) and sequence-specific primer (SSP) methodology; this compares to the 1 to 3 weeks turnaround time in completing four digit molecular HLA typing. Additionally, four-digit results can frequently be obtained using the SSO and qPCR methodology [23, 24]. Calculation of the number of eplet mismatches by serological antigen conversion appears to be an acceptable alternative, but several groups have reported discrepancies between HLA antigens defined by serological typing and PCR-SSP due to false negative or false positive reactivities and incomplete typing due to lack of serological reagents or assignment of broad antigens only [25–28]. Our study has further highlighted the major limitations and inaccuracy of serological-determined eplet mismatches and therefore is not an acceptable HLA typing method for calculating eplet mismatches.

There are several notable limitations. Firstly, our donor/recipient population was predominantly of Caucasian ethnicity. The conversions to four-digit alleles from two-digit HLA typing are performed using the catalogue of CWD alleles, derived largely from Caucasian population, and therefore patients or donors who were not Caucasian such as indigenous patients may have had an incorrect allele assigned [29]. As a result, the generalizability of these results to other heterogeneous populations where common alleles may not be present is unclear (and will need to be validated) and therefore, website such as National Marrow Donor Program (NMDP; www.haplostats.org) will need to be utilized as it provides an easy-to-use interface to estimate the most likely allelic typing based on haplotype frequency in different ethnic groups. However, there is little/no haplotype frequency data for the Australian indigenous population and any population comprising of mixed ethnicity groups further complicates haplotype assignment. Furthermore, HLA-DP was excluded in this analysis because serological or two-digit molecular HLA typing at the HLA-DP locus cannot be used to determine four-digit typing at this locus, which could potentially have underestimated the possible level of mismatching due to differences at DPA1 and DPB1 loci. However, low to intermediate HLA typing methods commonly define allele groups and some newer analysis software, such as those available with qPCR (Linkage Biosciences Inc) express HLA-DP antigens in epitope groups, allowing for a more accurate determination of eplet mismatches. In addition HLA-DQA, DRB 3/4/5 HLA typings were not included and additional mismatches at these loci may also be present. Rapid progress in HLA typing methods is likely to overcome such limitations in the near future potentially facilitating eplet-based deceased donor matching. In addition, this study only evaluates total number of eplet mismatches and does not differentiate between immunogenic versus non-immunogenic eplet mismatches.

## MATERIALS AND METHODS

### Study population

A retrospective cohort of 264 end-stage kidney disease patients who had either received a live or deceased donor kidney transplant between June 2003 and October 2007 in Western Australia, and their corresponding matched donors were included in this study. The human research ethics committees of the three tertiary hospitals approved the study, with written informed consents obtained from all patients.

### HLA-typing

Four-digit HLA-typing at class I (HLA-A, -B and -C) and class II (HLA-DPB1, -DQB1 and -DRB1) loci for all donors and recipients was performed using in-house Sanger sequencing-based HLA typing [30]. Molecular two-digit HLA typing was obtained using the two-digit information from Sanger sequencing. The two-digit typing was converted into four-digit typing using the catalogue of common and well documented (CWD) alleles.

Serological two-digit typing at HLA-A, -B and –DR loci was performed by complement-dependent cytotoxicity (CDC) technique using commercial monoclonal antibody trays (One Lambda Inc, California, United States). HLA-C and -DQ antigens were determined using the most commonly found linkage disequilibrium with HLA-B and -DR antigens respectively as published on http://www.ctht.info/resourceslinks.htm. The two-digit typing was converted into four-digit typing using the catalogue of CWD alleles. All serological and molecular HLA typing was performed at the Department of Clinical Immunology, Perth, Western Australia.

### Calculation of the number of eplet mismatches

The number of eplet mismatches for each donor/recipient pair at both class I and class II loci was calculated using HLAMatchmaker (Version 2.1; available from: www.hlamatchmaker.net). The HLAMatchmaker program requires the entry of an allele-level HLA type. However, when the allele-level HLA type is not present, the conversion to four-digit alleles was based on the frequency of the alleles in our population for a given haplotype. The conversion of two-digit serological or two-digit molecular typing to four-digit allelic typing was carried out according to the panel of CWD alleles derived from a number of populations worldwide [29]. For indigenous patients, the conversion from two to four digit typing was according to the CWD Caucasian HLA alleles. There are currently no CWD HLA alleles for the indigenous population. Four-digit molecular typing was directly entered into the HLAMatchmaker program without requiring conversion. HLA-DP alleles can only be determined using molecular typing methods with no serological equivalent. Furthermore, due to the nomenclature of HLA-DP alleles, two-digit molecular typing results in a high degree of unresolvable ambiguities and for this reason HLA-DP was not included in this study.

### Statistical analysis

Baseline characteristics of the cohort were described as mean (standard deviation [SD]), number (proportion) or as median (interquartile range [IQR]). Four-digit molecular typing was considered the gold standard (referent) in all analyses. Evaluation of reliability of measurements was assessed using intra-class correlation (expressed as correlation coefficient with 95% confidence intervals [95% CI]) to establish the consistency and absolute agreement in the number of eplet mismatches calculated by serological or two-digit molecular typing methods compared to four-digit molecular typing method. The intraclass correlation coefficient is a measure of the reliability of measurements (i.e. number of eplet mismatches). Consistency and absolute agreement are derived from the intraclass correlation coefficients when the systematic differences between measurements for all donor/recipient pairs are considered irrelevant or relevant, respectively. Bland Altman plots were constructed to show the average of the differences and limits of agreement (0 ± 1.96 SD) in the number of calculated class I and II eplet mismatches between the serological or two-digit molecular typing methods and four-digit molecular typing method. The proportion of donor/recipient pairs with a difference of 0, 1–5, 6–10, 11–20 and >20 eplet mismatches at both class I and II loci between serological or two-digit molecular typing methods and four-digit molecular typing method was determined.

## CONCLUSIONS

We have shown that in a predominantly Caucasian cohort, two-digit alleles converted to four-digit alleles reliably calculate the number of eplet mismatches at both class I and II loci compared to four-digit molecular HLA typing method. These results suggest that two-digit molecular HLA typing may be sufficient if the allocation of donor kidneys evolved to include eplet mismatches in the allocation algorithm. Even though the cost of four digit molecular HLA typing has substantially reduced to be comparable to two digit typing method, the main impediment of four digit typing in deceased donor kidney allocation remains the slow turnaround time. However, it must be emphasized that experienced tissue typists using appropriate resources that are applicable to the population of interest should undertake the conversion of two-digit alleles to 4-digit alleles.

## SUPPLEMENTARY MATERIALS FIGURES



## Abbreviations

ANZDATA: Australia and New Zealand Dialysis and Transplant
ANOVA: analysis of variance
CDC: complement-dependent cytotoxicity
CWD: common and well documented
CI: Confidence interval
HLA: human leukocyte antigen
IQR: interquartile range
PCR: Polymerase chain reaction
qPCR: quantitative PCR
SD: standard deviation
SSO: sequence specific oligonucleotides
SSP: sequence specific primers

## References

[1] Duquesnoy RJ, Marrari M (2002). HLAMatchmaker: a molecularly based algorithm for histocompatibility determination. II. Verification of the algorithm and determination of the relative immunogenicity of amino acid triplet-defined epitopes. Hum Immunol.

[2] Duquesnoy RJ (2002). HLAMatchmaker: a molecularly based algorithm for histocompatibility determination. I. Description of the algorithm. Hum Immunol.

[3] Duquesnoy RJ, Askar M (2007). HLAMatchmaker: a molecularly based algorithm for histocompatibility determination. V. Eplet matching for HLA-DR, HLA-DQ, and HLA-DP. Hum Immunol.

[4] Duquesnoy RJ, Marrari M, Mulder A, Sousa L, da Silva AS, do Monte SJ (2014). First report on the antibody verification of HLA-ABC epitopes recorded in the website-based HLA Epitope Registry. Tissue Antigens.

[5] Duquesnoy RJ, Marrari M, Tambur AR, Mulder A, Sousa L, da Silva AS, do Monte SJ (2014). First report on the antibody verification of HLA-DR, HLA-DQ and HLA-DP epitopes recorded in the HLA Epitope Registry. Hum Immunol.

[6] Wiebe C, Pochinco D, Blydt-Hansen TD, Ho J, Birk PE, Karpinski M, Goldberg A, Storsley LJ, Gibson IW, Rush DN, Nickerson PW (2013). Class II HLA epitope matching-A strategy to minimize de novo donor-specific antibody development and improve outcomes. Am J Transplant.

[7] Sapir-Pichhadze R, Tinckam K, Quach K, Logan AG, Laupacis A, John R, Beyene J, Kim SJ (2015). HLA-DR and -DQ eplet mismatches and transplant glomerulopathy: a nested case-control study. Am J Transplant.

[8] Claas FH, Witvliet MD, Duquesnoy RJ, Persijn GG, Doxiadis II (2004). The acceptable mismatch program as a fast tool for highly sensitized patients awaiting a cadaveric kidney transplantation: short waiting time and excellent graft outcome. Transplantation.

[9] Duquesnoy RJ, Witvliet M, Doxiadis II, de Fijter H, Claas FH (2004). HLAMatchmaker-based strategy to identify acceptable HLA class I mismatches for highly sensitized kidney transplant candidates. Transplant International.

[10] Sypek MP, Alexander SI, Cantwell L, Ierino FL, Ferrari P, Walker AM, Kausman JY (2017). Optimizing Outcomes in Pediatric Renal Transplantation Through the Australian Paired Kidney Exchange Program. Am J Transplant.

[11] Duquesnoy RJ (2006). A structurally based approach to determine HLA compatibility at the humoral immune level. Human Immunology.

[12] Duquesnoy RJ (2011). Antibody-reactive epitope determination with HLAMatchmaker and its clinical applications. Tissue Antigens.

[13] Duquesnoy RJ, Marrari M (2009). Correlations between Terasaki’s HLA class I epitopes and HLAMatchmaker-defined eplets on HLA-A, -B and -C antigens. Tissue Antigens.

[14] Wiebe C, Nevins TE, Robiner WN, Thomas W, Matas AJ, Nickerson PW (2015). The Synergistic Effect of Class II HLA Epitope-Mismatch and Nonadherence on Acute Rejection and Graft Survival. Am J Transplant.

[15] Lim WH, Chadban SJ, Clayton P, Budgeon CA, Murray K, Campbell SB, Cohney S, Russ GR, McDonald SP (2012). Human leukocyte antigen mismatches associated with increased risk of rejection, graft failure, and death independent of initial immunosuppression in renal transplant recipients. Clin Transplant.

[16] Kosmoliaptsis V, Gjorgjimajkoska O, Sharples LD, Chaudhry AN, Chatzizacharias N, Peacock S, Torpey N, Bolton EM, Taylor CJ, Bradley JA (2014). Impact of donor mismatches at individual HLA-A, -B, -C, -DR, and -DQ loci on the development of HLA-specific antibodies in patients listed for repeat renal transplantation. Kidney Int.

[17] Duquesnoy RJ (2017). Should epitope-based HLA compatibility be used in the kidney allocation system?. Hum Immunol.

[18] Do Nguyen HT, Wong G, Chapman JR, McDonald SP, Coates PT, Watson N, Russ GR, D'Orsogna L, Lim WH (2016). The association between broad antigen HLA mismatches, eplet HLA mismatches and acute rejection after kidney transplantation. Transplantation Direct.

[19] Hosomichi K, Shiina T, Tajima A, Inoue I (2015). The impact of next-generation sequencing technologies on HLA research. J Hum Genet.

[20] Tambur AR, Rosati J, Roitberg S, Glotz D, Friedewald JJ, Leventhal JR (2014). Epitope analysis of HLA-DQ antigens: what does the antibody see?. Transplantation.

[21] Duquesnoy RJ, Kamoun M, Baxter-Lowe LA, Woodle ES, Bray RA, Claas FH, Eckels DD, Friedewald JJ, Fuggle SV, Gebel HM, Gerlach JA, Fung JJ, Middleton D (2015). Should HLA mismatch acceptability for sensitized transplant candidates be determined at the high-resolution rather than the antigen level?. Am J Transplant.

[22] Heidt S, Witvliet MD, Haasnoot GW, Claas FH (2015). The 25th anniversary of the Eurotransplant Acceptable Mismatch program for highly sensitized patients. Transpl Immunol.

[23] Koehler RN, Walsh AM, Sanders-Buell EE, Eller LA, Eller M, Currier JR, Bautista CT, Wabwire-Mangen F, Hoelscher M, Maboko L, Kim J, Michael NL, Robb ML (2010). High-throughput high-resolution class I HLA genotyping in East Africa. PloS One.

[24] Testi M, Andreani M (2015). Luminex-Based Methods in High-Resolution HLA Typing. Methods Mol Med.

[25] Yu N, Ohashi M, Alosco S, Granja C, Salazar M, Hegland J, Yunis E (1997). Accurate typing of HLA-A antigens and analysis of serological deficiencies. Tissue Antigens.

[26] Bozon MV, Delgado JC, Selvakumar A, Clavijo OP, Salazar M, Ohashi M, Alosco SM, Russell J, Yu N, Dupont B, Yunis EJ (1997). Error rate for HLA-B antigen assignment by serology: implications for proficiency testing and utilization of DNA-based typing methods. Tissue Antigens.

[27] Otten HG, Tilanus MG, Barnstijn M, van Heugten JG, de Gast GC (1995). Serology versus PCR-SSP in typing for HLA-DR and HLA-DQ: a practical evaluation. Tissue Antigens.

[28] Mytilineos J, Christ U, Lempert M, Opelz G (1997). Comparison of typing results by serology and polymerase chain reaction with sequence-specific primers for HLA-Cw in 650 individuals. Tissue Antigens.

[29] Mack SJ, Cano P, Hollenbach JA, He J, Hurley CK, Middleton D, Moraes ME, Pereira SE, Kempenich JH, Reed EF, Setterholm M, Smith AG, Tilanus MG (2013). Common and well-documented HLA alleles: 2012 update to the CWD catalogue. Tissue Antigens.

[30] Sayer D, Whidborne R, Brestovac B, Trimboli F, Witt C, Christiansen F (2001). HLA-DRB1 DNA sequencing based typing: an approach suitable for high throughput typing including unrelated bone marrow registry donors. Tissue Antigens.

