# Supplementary material for: Correlation and agreement between eplet mismatches calculated using serological, low-intermediate and high resolution molecular human leukocyte antigen typing methods
Source: Oncotarget. 2018 Feb 1;9(17):13116–24. doi: 10.18632/oncotarget.24349 (PMC5862565; doi:10.18632/oncotarget.24349)
Supplement: Supplementary file 1 [file oncotarget-09-13116-s001.pdf]

Correlation and agreement between eplet mismatches calculated using serological, low-intermediate and high resolution molecular human leukocyte antigen typing methods

SUPPLEMENTARY MATERIALS

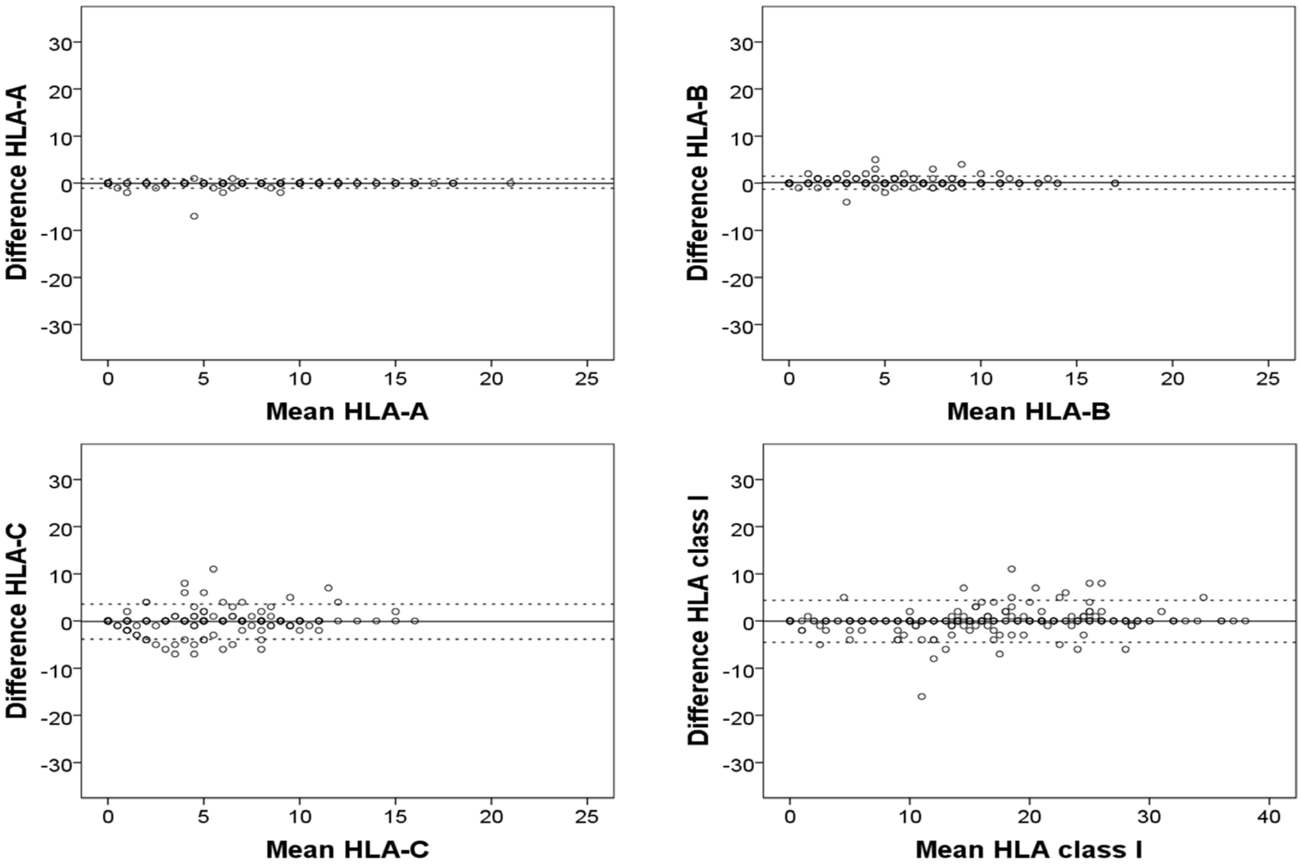

Supplementary Figure 1: Bland-Altman plots showing the mean differences and 95% limits of agreements between the number of eplet mismatches at the class I locus (i.e. human leukocyte antigen [HLA]-A, -B, -C and total class I) calculated by serological compared to four-digit high-resolution molecular HLA typing methods. Each open circle represents the estimated mean difference in the calculated number of eplet mismatches between serological and four-digit (referent) molecular HLA typing methods for each donor/recipient pair in the cohort. The continuous line represents a mean difference of 0 eplet mismatch between the two HLA typing methods, with the discontinuous lines representing a mean difference of 1.96 standard deviations above and below a difference of 0 eplet mismatch.

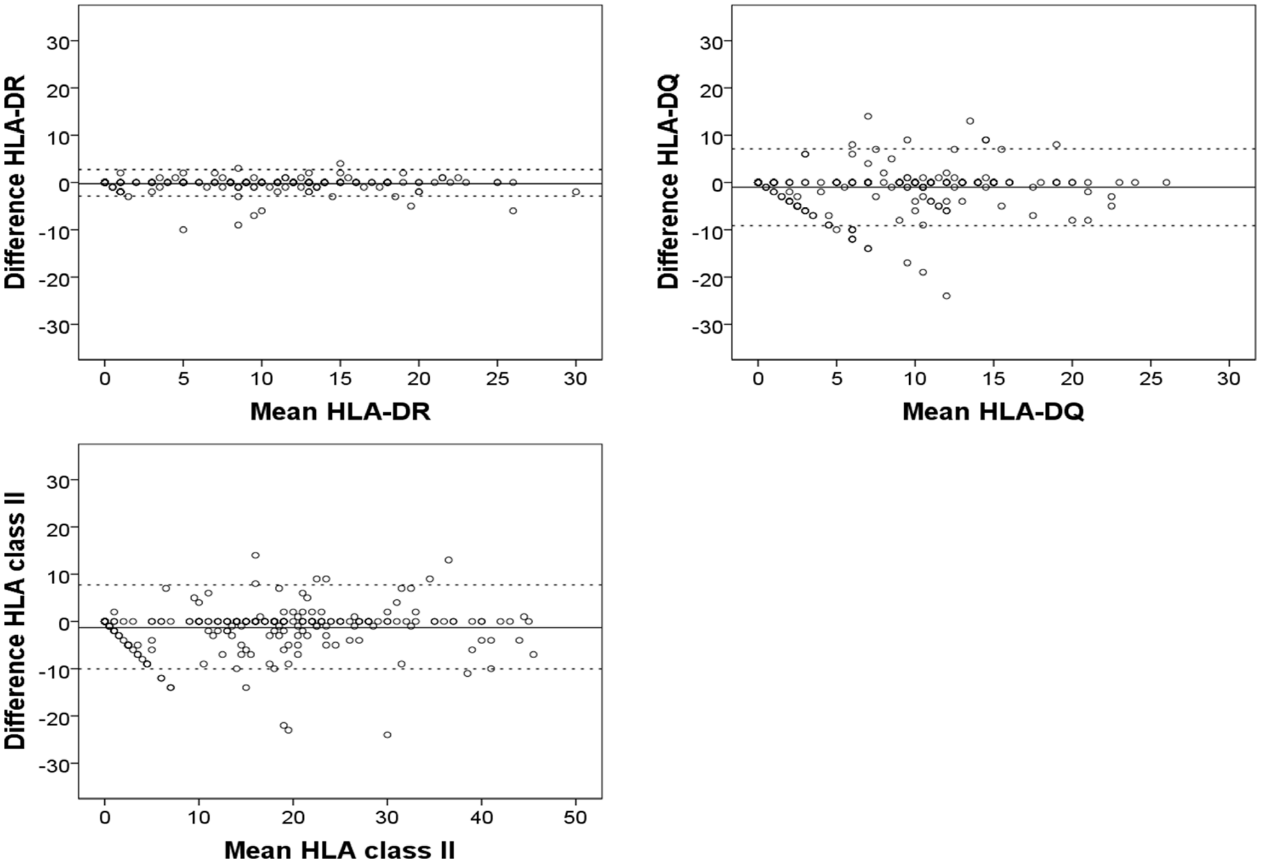

Supplementary Figure 2: Bland-Altman plots showing the mean differences and 95% limits of agreements between the number of eplet mismatches at the class II locus (i.e. human leukocyte antigen [HLA]-DR, -DQ and total class II excluding HLA-DP) calculated by serological compared to four-digit high-resolution molecular HLA typing methods. Each open circle represents the estimated mean difference in the calculated number of eplet mismatches between serological and four-digit (referent) molecular HLA typing methods for each donor/recipient pair in the cohort. The continuous line represents a mean difference of 0 eplet mismatch between the two HLA typing methods, with the discontinuous lines representing a mean difference of 1.96 standard deviations above and below a difference of 0 eplet mismatch.
